# Gaining access to the sterically occluded CD4-induced epitopes

**DOI:** 10.1186/1742-4690-9-S2-P80

**Published:** 2012-09-13

**Authors:** M Mengistu

**Affiliations:** 1 University of Maryland School of Medicine, Baltimore, MD, USA

## Background

A preventive vaccine is potentially the most effective way to control the HIV pandemic. Such a vaccine needs to successfully harness humoral immunity and produce cross-reactive anti-envelope antibodies that mediate direct virus neutralization and/or Fc receptor-dependent killing. The targeted HIV envelope spikes are covered by a glycan shield, which masks most of the surface from the humoral immune system, leaving very few sites that are antigenic. Among the vulnerable sites of HIV Env are intermediate structures formed after gp120 interaction with target CD4+ cell. The capacity of these CD4i antibodies to carry out their functions in clearing HIV infection is dependent on the timing, duration and extent of cognate epitope exposure during the attachment and entry processes.

## Methods

We employed confocal microscopy to visualize the temporal appearance and disappearance of CD4i epitopes during HIV-1 JRFL – TZM-bl cell interaction. We also examined the location of these exposed epitopes with ~20nm precision using super resolution microscopy.

## Results

We find that CD4i epitopes recognized by A32, 17b, and C11 were exposed on HIV-1JRFL within 5 minutes of interaction with TZM-bl cells, and persisted up to 60 minutes. 3D examination of confocal images revealed that these epitopes were exposed at sites distal to the virus – cell interface. CD4i epitope exposure was greatly reduced on mutant HIV-1 JRFL with a defective virus matrix (MA) as it interacts with TZM-bl cells.

## Conclusion

CD4i antibodies are thought to be sterically occluded from the virus – cell interface. Our results show that these epitopes appear distal to this site, where they can be accessed by antibodies involved in humoral and/or cell-mediated immunity. HIV Env gp120 engagement of target cell CD4 led to perturbations of virus MA that resulted in CD4i epitope exposure on other spikes of the virus away from gp120 – CD4 contact points.

